# Clinical practice guidelines for duodenal cancer 2021

**DOI:** 10.1007/s00535-022-01919-y

**Published:** 2022-10-19

**Authors:** Kenji Nakagawa, Masayuki Sho, Mitsuhiro Fujishiro, Naomi Kakushima, Takahiro Horimatsu, Ken-ichi Okada, Mikitaka Iguchi, Toshio Uraoka, Motohiko Kato, Yorimasa Yamamoto, Toru Aoyama, Takahiro Akahori, Hidetoshi Eguchi, Shingo Kanaji, Kengo Kanetaka, Shinji Kuroda, Yuichi Nagakawa, Souya Nunobe, Ryota Higuchi, Tsutomu Fujii, Hiroharu Yamashita, Suguru Yamada, Yukiya Narita, Yoshitaka Honma, Kei Muro, Tetsuo Ushiku, Yasuo Ejima, Hiroki Yamaue, Yasuhiro Kodera

**Affiliations:** 1 The Japan Duodenal Cancer Guideline Committee, 840 Shijo-cho, Kashihara, Nara 634-8522 Japan; 2 Department of Surgery, Nara Medical University, 840 Shijo-cho, Kashihara, Nara 634-8522 Japan

**Keywords:** Duodenal cancer, Clinical practice guidelines, Treatment, The Japan duodenal cancer guideline committee

## Abstract

Duodenal cancer is considered to be a small intestinal carcinoma in terms of clinicopathology. In Japan, there are no established treatment guidelines based on sufficient scientific evidence; therefore, in daily clinical practice, treatment is based on the experience of individual physicians. However, with advances in diagnostic modalities, it is anticipated that opportunities for its detection will increase in future. We developed guidelines for duodenal cancer because this disease is considered to have a high medical need from both healthcare providers and patients for appropriate management. These guidelines were developed for use in actual clinical practice for patients suspected of having non-ampullary duodenal epithelial malignancy and for patients diagnosed with non-ampullary duodenal epithelial malignancy. In this study, a practice algorithm was developed in accordance with the Minds Practice Guideline Development Manual 2017, and Clinical Questions were set for each area of epidemiology and diagnosis, endoscopic treatment, surgical treatment, and chemotherapy. A draft recommendation was developed through a literature search and systematic review, followed by a vote on the recommendations. We made decisions based on actual clinical practice such that the level of evidence would not be the sole determinant of the recommendation. This guideline is the most standard guideline as of the time of preparation. It is important to decide how to handle each case in consultation with patients and their family, the treating physician, and other medical personnel, considering the actual situation at the facility (and the characteristics of the patient).

## Introduction

Duodenal cancer, a representative rare cancer in gastrointestinal malignancies, is considered to be a small intestine carcinoma clinicopathologically; however, in Japan, there are no established guidelines for its treatment based on sufficient scientific evidence, and evidence, such as epidemiological data and phase III clinical trials that serve as the basis for such guidelines, are also lacking. Therefore, in daily clinical practice, its treatment is based on the experience of individual physicians and is similar to that of gastric and colorectal cancers. However, with advances in diagnostic modalities, such as gastrointestinal endoscopy and imaging, it is anticipated that opportunities for its detection will increase in future. We started to develop guidelines for management of duodenal cancer because this disease is considered to have high health-care demand by both healthcare providers and patients to provide appropriate medical care to patients. This guideline was compiled for patients with suspected non-ampullary duodenal epithelial malignancies (including adenomas and intra-mucosal carcinomas) and patients diagnosed with non-ampullary duodenal epithelial malignancies. The guidelines were developed for use in actual clinical practice, without limiting the gender or age of the target population.

**Fig. 1:**
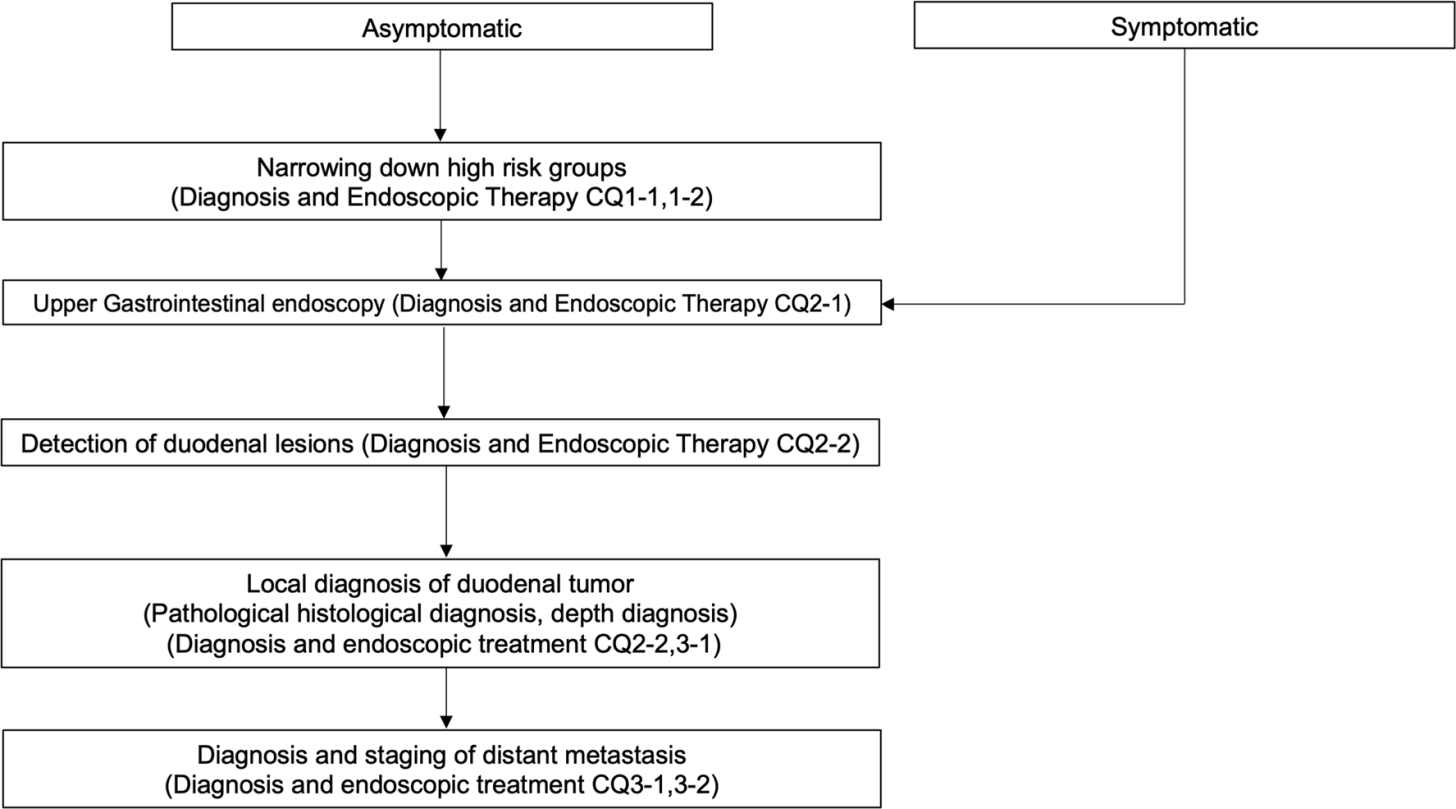
Diagnosis algorithm of duodenal cancer. *CQ* clinical question

**Fig. 2:**
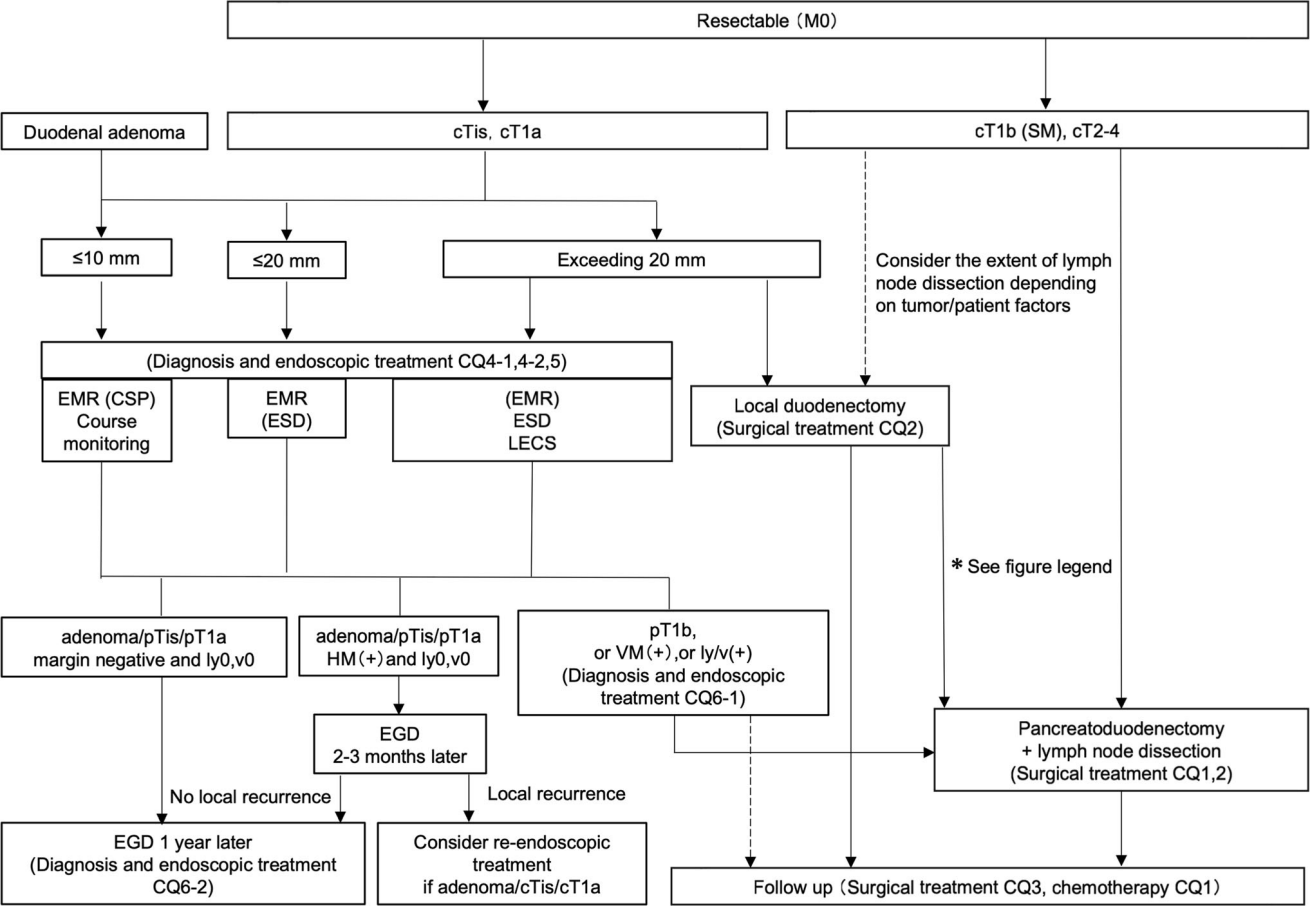
Treatment algorithm of resectable duodenal cancer. Although there is no evidence for additional surgery after local resection of the duodenum, a comprehensive decision an overall decision should be made whether to perform pancreato-duodenectomy plus lymph node dissection, taking into account pathological tumor and patient factors such as vascular invasion and residual cancer. *Tis* tumor confined to the lamina propria, *SM* tumor confined to the submucosa, *CQ* clinical question, *EMR* endoscopic mucosal resection, *CSP* cold snare polypectomy, *ESD* endoscopic submucosal dissection, *LECS* laparoscopy and endoscopy cooperative surgery, *HM* horizontal margin, *VM* vertical margin, *EGD* esophagogastroduodenoscopy

**Fig. 3:**
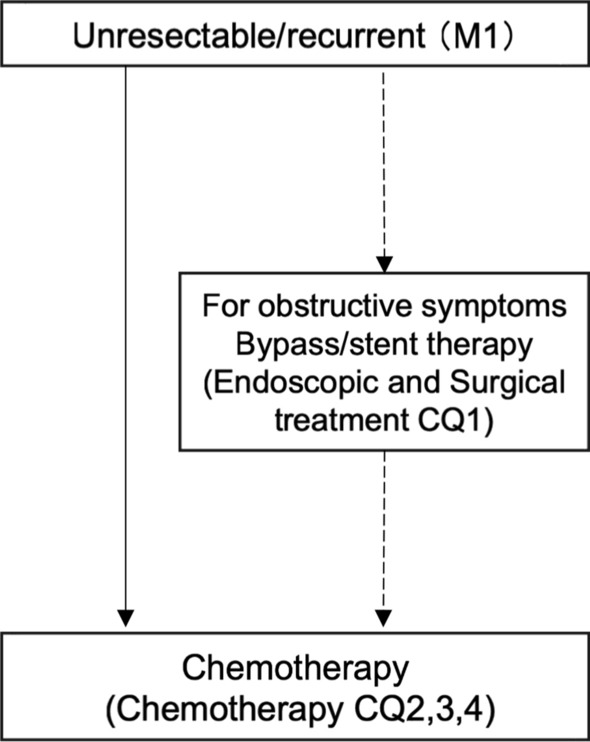
Treatment algorithm of un-resectable duodenal cancer. *CQ* clinical question

In this study, the practice guideline was developed in accordance with the Minds Practice Guideline Development Manual 2017 [1]. In accordance with the manual, we developed a practice algorithm (Table 1, Figs. 1, 2 and 3) and formulated Clinical Questions (CQs) for each area of epidemiology, diagnosis, endoscopic treatment, surgical treatment, and drug (chemo- and radiation)-therapy. An exemplary method was used to conduct a literature search using PubMed and medical journals, followed by a systematic review, draft recommendations, and a vote on the recommendations. For all CQs, we established relevant keywords and conducted an exhaustive primary screening of English and Japanese articles from 1945 to December 2018 (PubMed, The Cochrane Library) and from 1983 to December 2018 (Central Journal of Medicine). Reports from major international conferences and important papers were added by hand search as necessary, even outside the search period. The guideline development committee members and cooperating members independently conducted a secondary screening of the literature after the search to determine the articles to be adopted, and a systematic review was conducted. For each important outcome included in each CQ, the evidence presented by individual articles were categorized by study design, evaluated at the literature level and in aggregates, and the certainty (strength) of evidence for the CQ was ultimately determined. For those studies that had the same study design and for which efficacy measures could be evaluated quantitatively, a quantitative systematic review was conducted independently. Regarding those for which quantitative evaluation was not possible, only qualitative systematic reviews were conducted to evaluate the logic, certainty, etc. from the context.

**Table1:** TNM classification of duodenal cancer, based on the UICC 8th edition of small intestine cancer

Degree of progression	TNM classification
Epithelial	Tis
Local	T1a (invades lamina propria/muscularis mucosa), T1b (submucosa)
	T2 (muscularis propria)
	T3 (involvement of submucosa/peri-muscular tissue)
Regional lymph node metastasis	N1-2
Proximal organ invasion	T4 (penetrates visceral peritoneum/invades other organs and structures)
Distant metastasis	M1

*UICC* Union for International Cancer Control

In the manual for developing medical practice guidelines, it is assumed that a draft recommendation is prepared based on available evidence, and that the recommendation is discussed thoroughly to determine the level of recommendation. However, duodenal cancer is a rare disease, and there is little evidence based on randomized controlled trials and many retrospective studies. Therefore, the decision was based in part on discussions and a majority vote of specialists in each field. For each CQ, “balance of benefits and harms,” “patient preference,” and “impact of resources” were also comprehensively judged, and judgments were made more in line with actual clinical practice so that decisions on recommendations were not influenced solely by the level of evidence. The committee comprised members from multiple disciplines, including internal medicine, surgery, radiology, and pathology, to minimize bias in opinions. Furthermore, all recommendation decisions were made by unanimous vote, with the exception of the chairperson and supervisory committee members, to emphasize consensus. Abstentions were allowed. Members with financial/academic conflicts of interest abstained from voting. The strength of the recommendation was based on the GRADE Grid method. After consensus meetings with the committee members, a draft of guidelines was written and uploaded on the websites of the Japanese Gastric Cancer Association Society of Gastroenterology, the Japanese Society of Hepato-Biliary-Pancreatic Surgery, the Japan Gastroenterological Endoscopy Society, the Japanese Society for Radiation Oncology, and the Japanese Society for Cancer of the Colon and Rectum from February 15 to March 1, 2021, for public hearing. Taking into account the comments received from public hearing and committee members, the final revision was developed. Finally, the guidelines written in Japanese were published in August 2021.

## Diagnosis and endoscopic treatment

**CQ1-1.** **Etiology**

- The number of cases of duodenal cancer is reported to be 3.0–3.7 per million population in North America and 2.9–4.3 per million population in Europe, showing a gradual increasing trend. According to data from the National Cancer Registry of Japan, 3005 cases of duodenal cancer were diagnosed in 2016, an extremely high gross incidence of 23.7 per million population (calculated based on a total population of 126,933,000). The age of onset is in the 60–70 years age range, with a slightly higher incidence in male individuals. In Europe and the United States, it is reported that 10–22% of cases are localized at the time of diagnosis, but in Japan, 56% of cases were localized in 2016, and about half of them were treated endoscopically.

(Background Question)

*Comment*: The incidence is reported to be 3.0–3.7 per million population in North America [2, 3] and 2.9–4.3 per million population in Europe [4–6]. According to data from the National Cancer Registry of Japan, 3005 cases of duodenal cancers were diagnosed in 2016, with an extremely high crude rate of 23.7 per million population (calculated based on a total population of 126,993,000). Although reports from Europe indicate that there seems to be almost no difference between male and female patients [4, 6], data from the Japanese cancer registry show that male patients tend to be 1.5 times more common than female patients. The disease has been reported to be more common in people in their 60 and 70 s, accounting for approximately 55% of this age group, and the incidence increases with age [2, 4]. Approximately 10–22% of all cases have been reported to remain localized to the duodenum at the time of detection [2, 4]. In Japan, 56.4% of cases remain localized to the duodenum, 5.6% involve regional lymph nodes, 15.8% involve distant metastasis, 8.6% involve invasion of surrounding organs, and 13.6% are unknown. Additionally, approximately 48.0% of tumors that remained in the duodenal region were treated endoscopically [7].

**CQ1-2.** **What are the risks of duodenal cancer?**

- There are no known risk factors for non-ampullary duodenal cancer other than familial adenomatous polyposis of the colon (FAP).

(Background Question)

*Comment*: A systematic review of small intestinal cancers, including duodenal cancer, in the general population with low risk of bias reported associations with smoking, alcohol consumption, and various diseases (FAP, Crohn’s disease, cholecystectomy, peptic ulcer, cystic fibrosis, etc.). However, compared to the general population, the risk ratio for duodenal cancer in patients with FAP is 330.8 (132.7–681.5) [8]. The cumulative incidence of duodenal cancer (including papillary carcinoma) has been reported to be 7.7% (3.5–16.5%) [9] at the age of 50 years, with a lifetime risk of 18% (8–28) [10]. In patients with FAP, the presence of duodenal adenoma (Risk ratio, RR 13.2 [1.6–107.2]) is a risk factor for duodenal cancer [9]. Spigelman’s classification is used as the clinicopathologic classification of duodenal adenomas in patients with FAP [11]. In a retrospective cohort study [10], Spigelman stage IV (RR 6.4 [2.7–15.2]) was reported as a risk factor for duodenal cancer. In another case–control study [12], Spigelman stage IV (Odds ratio, OR 4.9 [1.6–15.1]) was also reported as a risk factor. Among the factors comprising Spigelman’s classification, high grade dysplasia (OR 6.7 [1.7–26.5]) and maximum tumor diameter > 10 mm (OR 3.7 [1.1–12.1]) were reported to be associated factors. In this study, Spigelman stage IV (OR 10.7 [2.0–74.2]) was a risk factor, as was the case for non-ampullary carcinoma of the duodenum, and among its components, high grade dysplasia (OR 12.1 [1.8–81.0]) and maximum diameter > 10 mm (OR 8.8 [1.1–407.2]).

**CQ2-1.** **Are duodenal adenomas eligible for treatment?**

- Weak recommendation for treating non-ampullary duodenal adenomas.

(Recommendation: weak, 100% agreed, evidence level C)

*Comment*: Duodenal adenomas are divided into sporadic and familial forms. FAP is the most frequent familial form, and the Spigelman classification is usually used for duodenal adenomas associated with it. Furthermore, the indication for treatment is determined by scoring based on tumor number, size, histology, and atypia [11]. In this CQ, we performed a qualitative systematic review of sporadic duodenal adenomas [13–17]. We considered that histological atypia of non-ampullary duodenal adenomas increases with follow-up even if they are sporadic, and in a certain percentage of cases, the histological diagnosis after endoscopic treatment is more atypical than the biopsy diagnosis before endoscopic treatment.

**CQ2-2.** **How can adenoma and cancer in duodenal tumors be differentiated?**

- Although histological diagnosis by biopsy is the standard for differentiating adenomas from cancers, performing endoscopic diagnosis when endoscopic treatment is being considered is weakly recommended.

(Recommendation: weak, 100% agreed, evidence level C)

*Comment*: Generally, the standard method of differentiating benign from malignant tumors of the gastrointestinal tract is biopsy before treatment. Histological diagnosis is highly specific in differentiating adenomas from carcinomas in duodenal tumors, and is a standard diagnostic method as with other gastrointestinal tumors. However, several studies have shown that the correct diagnosis rate of endoscopic diagnosis, including magnifying endoscopy with narrow-band imaging, is also comparable or higher than that of biopsy diagnosis [18–23]. Endoscopic diagnosis can also be weakly recommended when considering endoscopic treatment, including the effects of fibrosis of the lesion caused by biopsy.

**CQ3-1.** **What is recommended to differentiate intra-mucosal from submucosal carcinoma?**

- Evaluation by endoscopic gross type and coloration is weakly recommended.

(Recommendation: weak, 100% agreed, evidence level C)

*Comment*: Endoscopic gross type and color are important to distinguish M (tumor confined to the lamina propria) from SM (tumor confined to the submucosa), and endoscopic ultrasound (EUS) may be a reference finding, as several cases of SM invasion have been reported [24–26]. Future case accumulation is desirable for the diagnosis using magnifying endoscopy as well as EUS.

**CQ3-2.** **What is recommended for diagnosis of distant metastases?**

- Imaging, including contrast-enhanced CT scan, is weakly recommended.

(Recommendation: weak, 100% agreed, evidence level C)

*Comment*: Only one retrospective case–control report that met the intent of the CQ was found in the literature [27]. To date, computed tomography (CT) scans have been reportedly used for evaluating intramural and extramural extension of the primary tumor, vascular invasion, peri-duodenal adipose tissue invasion, adjacent organ invasion, lymph nodes, and other organ metastasis. The selected papers also concluded that CT examination including contrast studies can evaluate metastasis to other organs, vascular invasion, and invasion to adjacent organs, and is useful in determining whether radical resection is feasible. However, there are no reports on positron emission tomography (PET) or magnetic resonance imaging (MRI) for duodenal cancer, and we consider these imaging tests to be effective in the diagnosis of distant metastasis, although further accumulation of case reports is desirable.

**CQ4-1.** **What are the indications for various endoscopic treatments for duodenal neoplasms?**

- Although polypectomy, EMR, ESD, LECS, etc. have been performed, the indication criteria for various treatment methods are unclear.

(Background Question)

*Comment*: A paper on a survey of 13 Japanese institutions regarding endoscopic treatment of superficial non-ampullary duodenal epithelial tumor (SNADET) is available [28]. Although the survey has the largest number (1397) of cases in a Japanese paper, carcinoid and Brunner’s adenoma/hyperplasia were included in the target lesions, and it was not possible to evaluate the outcome of SNADET alone. Furthermore, no description of lesion size or indications for each treatment (endoscopic mucosal resection; EMR, endoscopic submucosal dissection; ESD, cold polypectomy; CP, underwater EMR; U-EMR, laparoscopy and endoscopy cooperative surgery; LECS) was provided, making it difficult to compare treatment outcomes. There were no randomized controlled trials of endoscopic treatment of SNADET, and most reports were retrospective observational studies. Furthermore, the reality of endoscopic treatment differs between Japan and other countries. Outside of Japan, EMR is the treatment of choice even for large tumors, and piecemeal EMR is often used, while all ESD reports are from Japan, where larger tumors are treated compared to EMR, and high en bloc and R0 resection rates are reported. The rate of recurrence was 0% [29–32]. However, the incidence of incidental adverse events was higher with ESD. The criteria for selecting EMR and ESD are currently based on the actual conditions of endoscopic treatment at each institution. Although the long-term prognosis of CP and U-EMR is unknown, it was suggested that CP and U-EMR may be effective treatment options with few complications for lesions with small tumor diameters, as there were no cases of perforation or bleeding [33, 34]. There were no observational studies of LECS and Argon plasma coagulation with more than 30 cases. From the above, it was considered difficult to establish uniform criteria for treatment methods.

**CQ4-2.** **What are the endoscopist and facility requirements for various types of endoscopic procedures?**

- Although there are no clear requirements for endoscopist and facilities, performance of ESD by endoscopist and facilities that are skilled in the technique is weakly recommended.

(Recommendation: weak, 100% agreed, evidence level C)

*Comment*: Although the incidence of adverse events during duodenal ESD has decreased over time, it is still higher than ESD for other organs, and there are reports of expert-led ESD in high-volume centers [28–30, 32, 35, 36], but there are only few reports from small and medium-sized centers.

There are no reports of LECS with a large number of cases. Although EMR has a lower incident rate compared to ESD, emergency surgery has been reported in some cases, and the procedure by novice endoscopist should be avoided. There were few reports from small and medium-sized facilities as an institutional requirement, but we judged that there was insufficient evidence to institutional requirements. Although the incidences of adverse events during cold snare polypectomy, cold forceps polypectomy, and U-EMR are low, and relatively safe procedures can be performed, discussions based on the R0 resection rate and other factors are also necessary. Therefore, there is little rationale for setting specific endoscopist and facility requirements.

**CQ5.** **Are prophylactic measures recommended after endoscopic treatment of superficial non-ampullary duodenal epithelial tumors?**

- Performance of prevention measures for adverse events during duodenal EMR and ESD, including mucosal suture and wound covering with PGA sheets, is weakly recommended.

(Recommendation: weak, 100% agreed, evidence level C)

*Comment*: Specific methods for the prevention of adverse events during endoscopic treatment for superficial non-ampullary duodenal tumors include clips/threaded clips or endoloops [29, 37–40], suturing with an Over-The-Scope Clip (OTSC) [32, 40, 41], or covering with a polyglycolic acid (PGA) sheet [37, 39], and laparoscopically assisted augmentation from the serosal side (so-called D-LECS) [42] were reported. While all these studies were retrospective, the incidence of adverse events was significantly reduced by taking various prevention measures. A quantitative systematic review by four editors [29, 33, 37, 39], in which comparisons were made with the target population, found that the risk was reduced by approximately 84%. However, OTSC, PGA sheets with fibrin glue are expensive. Fibrin glue is a blood product derived from donated blood and carries a low risk of infection. However, adverse events that occur after endoscopic treatment in the duodenum are often very serious, and from the viewpoint of the balance of benefits and harms, it is recommended that measures be taken to prevent late-onset adverse events.

**CQ6-1.** **What are the recommended criteria for surgical treatment after endoscopic treatment?**

- Additional surgery in cases of submucosal carcinoma and vascular invasion is weakly recommended.

(Recommendation: weak, 100% agreed, evidence level C)

*Comment*: Additional surgery is recommended for submucosal carcinoma because of the high risk of local recurrence and lymph node metastasis. Although the local recurrence rate is high in patients undergoing piecemeal resection, strict follow-up can be considered because subsequent endoscopic treatment is effective and shows a good prognosis [36, 43–48]. Although there were few reports on vascular invasion after endoscopic treatment, additional surgery was recommended based on surgical treatment cases [49].

**CQ6-2.** **Is endoscopic surveillance recommended after endoscopic treatment for early detection of local recurrence and metachronous lesions?**

- Endoscopic surveillance for local recurrence after endoscopic treatment is weakly recommended.

(Recommendation: weak, 100% agreed, evidence level C)

*Comment*: In the qualitative systematic review conducted in this study, no high-quality studies such as prospective studies that followed a defined surveillance methodology were identified; only retrospective studies were identified. No evidence was found on the detection of metachronous lesions, as well as on the interval and duration of surveillance [29, 36, 43, 48, 50–52]. The majority of the locally recurrent lesions detected by surveillance were controlled endoscopically, and no cause specific mortality was observed. However, surveillance costs less than surgery [52], considering the balance of benefits and harms, endoscopic surveillance for the detection of local recurrence after endoscopic treatment may be beneficial, as the mortality rate due to endoscopic complications was low at 0.001% [53]. Furthermore, a high recurrence rate has been reported in lesions that have been treated in a piecemeal fashion, and surveillance is especially desirable.

## Surgical treatment

**CQ1.** **Is lymph node dissection recommended in the surgical treatment of duodenal cancer?**

- Lymph node dissection is weakly recommended in the surgical treatment of duodenal cancer. However, lymph node dissection may be omitted for intra-mucosal lesions.

(Recommendation: weak, 96% agreed, evidence level D)

*Comment*: In previous retrospective studies in duodenal cancer, lymph node-positive cases were associated with significantly poorer prognosis [54–57]. Furthermore, numerous multivariate analyses have reported that lymph node metastasis is an independent prognostic factor along with progression, histologic differentiation, and vascular invasion [56, 58, 59]. Although there are not many reports on the frequency of lymph node metastasis according to tumor localization in the duodenum, in the first portion of duodenum, the lymph nodes in the subpyloric region (No. 6) and the posterior pancreatic head (No. 13), and in the descending part of the duodenum, the lymph nodes in the posterior pancreatic head (No. 13) and anterior pancreatic head (No. 17) are considered to be sentinel lymph nodes [60], and lymphatic flow in the transvers and ascending part of the duodenum is speculated to flow from the inferior pancreatoduodenal artery and upper jejunal artery into the lymphatic system around the superior mesenteric artery [61]. It is also possible that the preferred site of lymph node metastasis differs depending on localization [59]. Meanwhile, a study on the relationship between tumor depth and lymph node metastasis showed that the lymph node metastasis rate for submucosal carcinoma is 5–11%, and the frequency is even higher in the intrinsic muscle layer and deeper (MP; tumor invasion of the muscularis propria: 44%, SS; tumor invasion of the sub-serosa: 41%, SE; tumor invasion that is contiguous to or penetrates the serosa and is exposed to the peritoneal cavity /Sl; tumor invasion of adjacent structures: 73%). In addition, it is often reported that lymph node metastasis is not observed in intra-mucosal lesions [62–66].

However, the above reports only indicate the presence and frequency of lymph node metastasis. There is no evidence that shows that lymph node dissection for duodenal cancer contributes to a prolonged prognosis. There is also no evidence regarding the optimal extent of dissection or complications associated with lymph node dissection. Therefore, evidence is needed to discuss its balance of benefits and harms can be considered. Although it is necessary to mention that the body of clear evidence for this CQ is insufficient, peripheral lymph node dissection may be considered to the extent that it can be safely resected. If the lesion is deeper than the submucosa, a surgery with surrounding lymph node dissection, such as pancreato-duodenectomy, can be performed regardless of the location of the lesion in the duodenum. Only when surgical treatment is indicated and the lesion is judged to be intra-mucosal, a modified operation such as distal gastrectomy or partial duodenectomy may be considered, depending on the location of the lesion. Clinical indications are based on whether vital organ function is preserved, and performance status is maintained.

**CQ2.** **In consideration of the depth and site of occupancy, is performing a procedure other than pancreato-duodenectomy recommended?**

- In cases of duodenal cancer deeper below the submucosa, we weakly recommend that no procedure other than pancreato-duodenectomy be performed.

(Recommendation: weak, 79% agreed, evidence level C)

*Comment*: As discussed in CQ1, lymph node metastasis is reported to occur in duodenal cancer that extend deeper than the submucosal layer, and the frequency of lymph node metastasis increases as the depth of the disease progresses. For duodenal cancer in the submucosal layer or deeper, pancreato-duodenectomy is currently proposed as the standard procedure, taking tumor factors into consideration. However, several case series have reported that the 5-year postoperative survival rates of pancreato-duodenectomy and limited resection of the duodenum (including pancreas-sparing partial duodenectomy and local duodenectomy) for duodenal cancer are similar [49, 56–58, 67–69]; furthermore, the incidence of postoperative complications, such as operative mortality and pancreatic fistula, tend to be higher for pancreato-duodenectomy [58, 67, 70–73]. Considering these surgical results and the frequency of lymph node metastasis, local duodenal resection (including endoscopic treatment) without lymph node dissection can be selected for intra-mucosal cancer, regardless of the site of occupancy. In addition, the preferred site of lymph node metastasis may differ depending on the site of occupancy. Furthermore, the efficacy and safety of pancreato-duodenectomy for duodenal cancer have not been fully established. Therefore, it may be appropriate to choose a technique other than pancreato-duodenectomy, such as local resection of the duodenum with lymph node dissection proximal to the tumor, for duodenal cancers that extend deeper than the submucosa, taking tumor and patient-related factors into full consideration.

**CQ3.** **What follow-up is recommended for diagnosis of recurrence after surgical resection of duodenal cancer?**

- After surgical treatment of duodenal cancer, careful follow-up with various imaging tests is weakly recommended for the diagnosis of distant metastasis and local recurrence.

(Recommendation: weak, 100% agreed, evidence level C)

*Comment*: Based on the idea that early diagnosis of recurrence by periodic follow-up in other gastrointestinal cancers leads to appropriate subsequent treatment, we propose careful follow-up according to the actual situation of each case and facility.

## Endoscopic and surgical treatment

**CQ1.** **Is gastrointestinal anastomosis or endoscopic stenting recommended for un-resectable duodenal cancer with obstructive symptoms?**

- Gastrointestinal anastomosis and endoscopic stent insertion are weakly recommended when these procedures are expected to be effective.

(Recommendation: weak, 100% agreed, evidence level D)

*Comment*: Surgical gastric jejunal bypass and endoscopic stent insertion are expected to restore oral intake and improve quality of life, as well as extend survival due to the ability to endure chemotherapy and chemoradiation. However, to date, there have been no reports on these outcomes in detail in un-resectable duodenal cancer, and the evidence for this CQ is insufficient. However, based on actual clinical practice and reflecting the opinions of the guideline drafting committee members, a consensus was reached that gastrointestinal anastomosis and endoscopic stent insertion for un-resectable duodenal cancer with obstructive symptoms are weakly recommended if they are expected to be effective.

## Chemotherapy

**CQ1.** **Is perioperative adjuvant therapy recommended for small bowel cancer, including resectable duodenal cancer?**

- We weakly recommend against performing postoperative adjuvant therapy to treat resectable small bowel cancer.

(Recommendation: weak, 96% agreed, evidence level D).

*Comment*: A literature search for CQ identified 17 articles [57, 58, 71, 74–87]. There are no randomized controlled trials that compared surgery alone with perioperative adjuvant therapy in patients with small bowel cancer, including resectable duodenal cancer. All 17 articles are retrospective comparisons of outcomes between cases treated with surgery alone and cases treated with perioperative adjuvant therapy, using single/multicenter treatment cases or national clinical database (NCD) data, and no articles on preoperative adjuvant therapy were identified.

Although three studies [75, 80, 81] showed an overall survival benefit with adjuvant therapy, the remaining 14 concluded that “adjuvant therapy does not contribute to an overall survival benefit”. Of those showing an overall survival benefit with adjuvant therapy, only one has been studied with a sufficient sample size, and it was concluded that the benefit was particularly high in stage III patients.

However, three meta-analysis articles [57, 84, 87] all concluded that “adjuvant therapy does not contribute to prolonged survival”. The variability of the above results is due to the fact that this was a retrospective study, therefore, selection bias among the patients was likely, and there was a large variation in perioperative treatment among the reports of chemotherapy/radiotherapy/chemoradiotherapy. Furthermore, the chemotherapy regimens employed varied widely from report to report, which may have had an impact on the results. Randomized controlled trials using a uniform treatment regimen are needed to answer this CQ.

**CQ2.** **Are MSI, HER2, and** ***RAS*** **gene tests recommended for small bowel cancer, including un-resectable or recurrent duodenal cancer?**

- MSI testing is strongly recommended.

(Recommendation: strong, 96% agreed, evidence level B)

- We weakly recommend against performing HER2 and *RAS* gene tests.

(Recommendation: weak, 100% agreed, evidence level D)

*Comment*: Tests for mismatch-repair defects include the microsatellite instability (MSI) test, which examines differences in microsatellite length associated with abnormal repeat counts in microsatellite regions, and the mismatch-repair (MMR) proteins (MLH1, MSH2, MSH6, PMS2) for immunohistochemistry, and next-generation sequencers are available. In Japan, MSI-High should be confirmed when pembrolizumab is administered to patients with duodenal cancer. In this review, there was no difference in frequency according to the testing method [88–118]. See CQ4 for information on immune checkpoint inhibitors for MSI-High cases. Since there are a certain number of MSI-High cases in duodenal cancer, and the results of the MSI-High test are expected to be effective with pembrolizumab, MSI testing is strongly recommended when tissue biopsy can be performed safely.

However, no drug has been shown to be effective for HER2 and *RAS* gene testing, even based on test results, and the significance of these tests is not clear at this time.

**CQ3.** **Is systemic chemotherapy recommended for small bowel cancer, including un-resectable or recurrent duodenal cancer?**

- Systemic chemotherapy with pyrimidine fluoride and oxaliplatin is weakly recommended for small intestinal cancer, including un-resectable or recurrent duodenal cancer.

(Recommendation: weak, 100% agreed, evidence level D)

*Comment*: Only single-arm prospective [119] and retrospective studies [83, 120–137] have investigated whether systemic chemotherapy improves prognosis in patients with small bowel cancer, including un-resectable or recurrent duodenal cancer. There have been no randomized comparative studies with best supportive care, and the results are still unclear. In retrospective studies of first-line treatment, combination therapy with pyrimidine fluoride and oxaliplatin is the most commonly used regimen, and cisplatin, irinotecan, and gemcitabine have also been reported. In a report that compared the efficacy of different treatment regimens, for pyrimidine fluoride plus oxaliplatin combination therapy, the response rate was 34–42%, median progression-free survival was 6.9–8.2 months, and median overall survival was 17.8–22.2 months; in combination with pyrimidine fluoride and cisplatin, response rates ranged from 31 to 38%, median progression-free survival ranged from 3.8 to 4.8 months, and median overall survival ranged from 9.3 to 12.6 months; and for irinotecan plus pyrimidine fluoride combination therapy, the response rate was 9–25%, median progression-free survival was 5.6–6.0 months, and median overall survival was 9.4–10.6 months. Although randomized controlled trials using a uniform treatment regimen are needed to answer this CQ, based on the above results, combination therapy based on pyrimidine fluoride and oxaliplatin is recommended as primary therapy when systemic chemotherapy is administered.

**CQ4.** **Are immune checkpoint inhibitors recommended for small bowel cancer, including un-resectable or recurrent duodenal cancer?**

- Pembrolizumab alone is strongly recommended only for previously treated un-resectable or recurrent small bowel cancer, including duodenal cancer, with MSI-High or dMMR.

(Recommendation: strong, 92% agreed, evidence level B)

*Comment*: To date, there have been no phase III trials of immune checkpoint inhibitors for small bowel cancer. Four papers [138–141] have reported the efficacy of pembrolizumab in small bowel cancer, including solid tumors with MSI-High overall, and the overall response rate (ORR) of pembrolizumab monotherapy ranged from 0 to 71%. A phase II trial investigating the efficacy of pembrolizumab monotherapy in 40 previously treated patients with small bowel cancer (including 24 patients with duodenal cancer) has been reported [142]. The primary endpoint of ORR was 8% (95% CI, 2–20), and the primary endpoint could not be achieved. A total of 26 patients who underwent MSI testing had an ORR of 50% in MSI-High patients (*n* = 4) and 10% in patients without microsatellite instability (microsatellite stable) (*n* = 20).

The U.S. Food and Drug Administration (FDA) approved pembrolizumab for MSI-High or mismatch-repair deficient (dMMR) solid tumors in 2017 in the United States, and this drug was approved in Japan in December 2018. There are no reports of phase III trial results comparing pembrolizumab with existing chemotherapies for duodenal cancer. Although there are a small number of cases, the results suggest that pembrolizumab compares favorably with the response rate and safety of existing chemotherapy in the treatment of MSI-High or dMMR solid tumors. Considering that duodenal cancer with MSI-High or dMMR is a rare disease, pembrolizumab monotherapy is strongly recommended for duodenal cancer with MSI-High or dMMR.

## Conclusion

These guidelines are the most standardized guidelines at the time of their creation, and do not regulate the implementation of medical treatment methods that differ from the indications described in the guidelines. It is important to decide the treatment plan for each case based on discussions with the patient/family as well as the doctors and other medical staff involved in the treatment, while considering the actual capabilities of the facility (personnel, experience, equipment, etc.) and the characteristics of the patient (shared decision making). In duodenal cancer treatment, physicians should refer to these guidelines together with their patients and try to explain the position and details of each diagnosis and treatment method in a simple manner for the patient’s understanding. If a patient is to be treated differently from the treatment recommended in the guidelines, it is necessary to explain to the patient why the treatment is being chosen and to ensure that the patient fully understands the reasons.

## Appendix

Members of the Guidelines Committee who created and evaluated the “Clinical practice guidelines for Duodenal Cancer 2021” are listed below. Chair: Masayuki Sho (Department of Surgery, Nara Medical University, Nara, Japan) Members: Mitsuhiro Fujishiro (Department of Gastroenterology, Graduate School of Medicine, The University of Tokyo, Tokyo, Japan), Naomi Kakushima (Department of Gastroenterology, Graduate School of Medicine, The University of Tokyo, Tokyo, Japan), Ken-ichi Okada (Second Department of Surgery, School of Medicine, Wakayama Medical University, Wakayama, Japan), Takahiro Horimatsu (Department of Therapeutic Oncology, Graduate School of Medicine, Kyoto University, Kyoto, Japan), Mikitaka Iguchi (Second Department of Internal Medicine, Wakayama Medical University, Wakayama, Japan), Toshio Uraoka (Department of Gastroenterology and Hepatology, Graduate School of Medicine, Gunma University, Gunma, Japan), Motohiko Kato (Division of Research and Development for Minimally Invasive Treatment, Cancer Center, Keio University School of Medicine, Tokyo, Japan), Yorimasa Yamamoto (Department of Gastroenterology, Showa University Fujigaoka Hospital, Kanagawa, Japan), Toru Aoyama (Department of Surgery, Yokohama City University, Yokohama, Japan), Takahiro Akahori (Department of Surgery, Nara Medical University, Nara, Japan), Hidetoshi Eguchi (Department of Gastroenterological Surgery, Graduate School of Medicine, Osaka University, Osaka, Japan), Shingo Kanaji (Division of Gastrointestinal Surgery, Department of Surgery, Graduate School of Medicine, Kobe University, Kobe, Japan), Kengo Kanetaka (Department of Surgery, Graduate School of Biomedical Sciences, Nagasaki University, Nagasaki, Japan), Shinji Kuroda (Department of Gastroenterological Surgery, Okayama University Graduate School of Medicine, Dentistry and Pharmaceutical Sciences, Okayama, Japan), Kenji Nakagawa (Department of Surgery, Nara Medical University, Nara, Japan), Yuichi Nagakawa (Department of Gastrointestinal and Pediatric Surgery, Tokyo Medical University, Tokyo, Japan), Souya Nunobe (Department of Gastroenterological Surgery, Cancer Institute Hospital, Tokyo, Japan), Ryota Higuchi (Department of Surgery, Institute of Gastroenterology, Tokyo Women’s Medical University, Tokyo, Japan), Tsutomu Fujii (Department of Surgery and Science, Faculty of Medicine, Academic Assembly, University of Toyama, Toyama, Japan), Hiroharu Yamashita (Department of Digestive Surgery, Nihon University School of Medicine, Tokyo, Japan), Suguru Yamada (Department of Gastroenterological Surgery, Nagoya Central Hospital, Nagoya, Japan), Yukiya Narita (Department of Clinical Oncology, Aichi Cancer Center Hospital, Nagoya, Japan), Yoshitaka Honma (Department of Head and Neck, Esophageal Medical Oncology, National Cancer Center Hospital, Tokyo, Japan.), Kei Muro (Department of Clinical Oncology, Aichi Cancer Center Hospital, Nagoya, Japan), Tetsuo Ushiku (Department of Pathology, Graduate School of Medicine, The University of Tokyo, Tokyo, Japan), Yasuo Ejima (Department of Radiology, Dokkyo Medical University, Tochigi, Japan) Supervisors: Hiroki Yamaue (Second Department of Surgery, School of Medicine, Wakayama Medical University, Wakayama, Japan) and Yasuhiro Kodera (Department of Gastroenterological Surgery (Surgery II), Nagoya University Graduate School of Medicine, Nagoya, Japan).

## Abbreviations

CQ: Clinical question
FAP: Familial adenomatous polyposis
RR: Risk ratio
OR: Odds ratio
M: Tumor confined to the lamina propria
SM: Tumor confined to the submucosa
EUS: Endoscopic ultrasound
CT: Computed tomography
PET: Positron emission tomography
MRI: Magnetic resonance imaging
SNADET: Superficial non-ampullary duodenal epithelial tumor
EMR: Endoscopic mucosal resection
ESD: Endoscopic submucosal dissection
CP: Cold polypectomy
U-EMR: Underwater EMR
LECS: Laparoscopy and endoscopy cooperative surgery
PGA: Polyglycolic acid
OTSC: Over-The-Scope Clip
MP: Tumor invasion of the muscularis propria
SS: Tumor invasion of the subserosa
SE: Tumor invasion that is contiguous to or penetrates the serosa and is exposed to the peritoneal cavity
SI: Tumor invasion of adjacent structures
NCD: National clinical database
MSI: Microsatellite instability
MMR: Mismatch-repair
dMMR: Mismatch-repair deficient
ORR: Overall response rate
